# Spin-Transport Tuning of Individual Magnetic Mn-Salophen Molecule via Chemical Adsorption

**DOI:** 10.3390/molecules24091747

**Published:** 2019-05-06

**Authors:** Feifei Li, Jing Huang, Jianing Wang, Qunxiang Li

**Affiliations:** 1 School of Materials and Chemical Engineering, Anhui Jianzhu University, Hefei 230601, Anhui, China; lff@ahjzu.edu.cn; 2 Department of Chemical Physics, University of Science and Technology of China, Hefei 230026, Anhui, China; Jiawang1@mail.ustc.edu.cn; 3 Hefei National Laboratory for Physical Sciences at the Microscale & Synergetic Innovation Center of Quantum Information and Quantum Physics, University of Science and Technology of China, Hefei 230026, Anhui, China

**Keywords:** spin-filtering, spin switch, chemical adsorption, magnetism, electronic structure

## Abstract

Control over spin states at the single molecule level is a key issue in the emerging field of molecular spintronics. Here, we explore the chemical adsorption effect on the magnetic and spin-transport properties of individual magnetic molecule by performing extensive density functional theory calculations in combining with non-equilibrium Green’s function method. Theoretical results clearly reveal that the molecular magnetic moment of Mn-salophen can be effectively tuned by adsorbing F and CO on the central Mn cation, while the adsorbed NO molecule quenches the molecular magnetic moment. Without chemical adsorption, the currents through Mn-salophen molecular junction just show a little distinction for two spin channels, which agrees well with previous investigation. Remarkably, the conductive channel can be switched from the spin-up electrons to the spin-down electrons via adsorbing F and CO, respectively, and the corresponding two Mn-salophen molecular junctions with chemical modifications display nearly perfect spin-filtering effect. The observed spin switch and the predicted spin-filtering effect via chemical adsorption indicates that Mn-salophen holds potential applications in molecular spintronic devices.

## 1. Introduction

Molecular spintronic devices based on the magnetic molecule as the functional units, such as molecular rectifier, molecular switch, and molecular transistor, have already attracted a great deal of attention in recent years since it holds promise for the next generation of electronic devices with enhanced functionality and improved performance [1,2,3,4,5,6,7]. It is well known that the electronic structure, magnetism and transport property of magnetic molecules can be effectively altered by varies external stimulus, such as electric field, strain, and carrier doping [8,9,10]. Recently, several experimental and theoretical investigations have demonstrated that an efficient method, via chemical adsorption using small molecules, such as CO, NO, NH${}_{3}$, and O${}_{2}$ [11,12,13,14,15,16], to manipulate the spin states and then tune their transport properties of magnetic molecules. For example, Wäckerlin and his coworkers have shown that the attachment of a diatomic molecule (i.e., NO) to Co(II) tetraphenylporphyrin can efficiently quenches the molecular spin states in their experiments [17]. Kondo et al. performed theoretical simulations to examine the changes in electrical conductance of a single Fe-porphyrin molecular junction via connecting CO, NO, and O${}_{2}$ molecules to the Fe cation, and they found that these conductive molecular orbitals and the corresponding transmission spectra are significantly modified by the adsorption of diatomic ligand gases [18]. Clearly, understanding the chemical adsorption effect is crucial to gain control over the spin states and spin-transport properties of a given magnetic molecules.

Molecular spin filter has been demonstrated to be an important spintronic device with conducting for one spin direction and insulating for the opposite direction. Till now, nearly perfect spin-filtering effect has been observed in many molecular junctions based on various molecules, including MnCu single-molecule magnet [19,20], spin-crossover magnet Fe${}_{2}$ and Fe(II)-N${}_{4}$S${}_{2}$ complexes [21,22,23], metal-phthalocyanines (MPc, M = Mn, Fe, Co, Ni, Cu, Zn) [24,25], Fe-cyclooctatetraene [26], and Eu-cyclooctatetraene [27]. We note that Mn-salophen [N-N${}^{\prime }$-bis(salicylaldehyde)-1,2-phenylenediiminometal(II)] has been extensively studied on their electrochemical and spectroelectrochemical properties [28,29], but the investigation of their transport behavior is limited so far. Chen et al. investigated the spin transport properties of four *3d* transition metal(II) salophens (TM = Co, Fe, Ni and Mn), and found that only Co-salophen junction display robust spin-filtering effect [30].

Here, we revisit Mn-salophen and try to explore the chemical adsorption effect on the magnetic and spin-transport properties of Mn-salophen sandwiched between two Au(100) electrodes by performing extensive density functional theory calculations combining with non-equilibrium Green’s function method. According to the calculated molecular magnetic moments, we find that the spin states of Mn-salophen can be easily controlled via chemical adsorption. Remarkably, upon the F and CO adsorption on the central Mn cation, two molecular junctions can act as efficient spin filters, and the corresponding conductance is dominated by the spin-up and spin-down electrons, respectively. The observed nearly perfect spin-filtering effect and the switching between two spin channels implies that Mn-salophen is a promising candidate for designing molecular spintronic devices.

## 2. Computational Parameters and Method

Here, geometry optimizations, electronic structures and transport properties of Mn-salophen with F, CO, and NO chemical adsorptions are calculated by using ATK package [31,32,33], which combines DFT calculations with non-equilibrium Green’s function (NEGF) technique. This kind of DFT+NEGF method has been successfully used to explain experimental results [34]. The exchange and correlation contributions are described by the generalized gradient approximation (GGA) in the Perdew-Burke-Ernzerhof (PBE) form. The Troullier-Martins nonlocal pseudopotentials are used to model the interaction between ionic cores and valence electrons. In our calculations, we employ double-zeta plus polarized basis sets for all elements. To self-consistently solve the Poisson equation, the energy cutoff is set to be 150 Ry for the real-space grid. The spin-resolved transmission coefficients are calculated by

(1) $$T_{\sigma }\left(E,V\right)=Tr\left[\Gamma_{L}G_{\sigma }\Gamma_{R}G_{\sigma }^{+}\right],$$

here, $\Gamma_{L}$ ($\Gamma_{R}$) is the coupling matrix between the extended molecule and the left (right) electrode, G${}_{\sigma }$ is the spin-dependent retarded Green’s function of the extended molecule, and σ stands for the spin channel, using ↑ and ↓ label for the spin-up and spin-down electrons, respectively. Based on the Landauer-Büttiker formula, the current is determined by

(2) $$I\left(V\right)=\frac{e}{h}\int T_{\sigma }\left(E,V\right)\left[f\left(E-\mu_{L}\right)-f\left(E-\mu_{R}\right)\right]dE,$$

here, the f(E-$\mu_{L(R)}$) is the Fermi-Dirac function for the left and right electrodes with the chemical potential $\mu_{L(R)}$.

## 3. Results and Discussion

**Figure 1:**
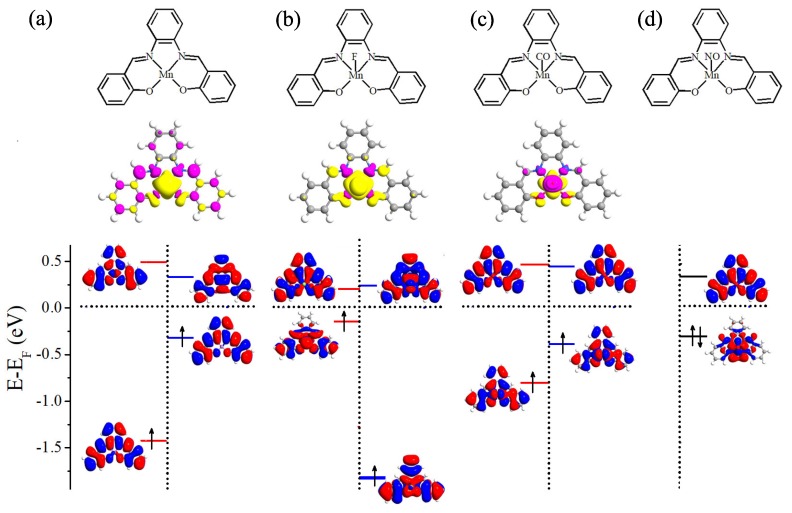
Schematic view of molecular geometric structure (top panel), spin density (middle panel), and the energy levels and the spatial distribution of the HOMO and LUMO (bottom panel). Here, (**a**) free Mn-salophen molecule, (**b**–**d**) Mn-salophen molecule with F, CO, and NO adsorptions. The short-dotted horizontal lines stand for the Fermi level, which is defined as the center place of the HOMO-LUMO gap of the spin-up or spin-down channel.

Before examining the chemical adsorption effect, we perform benchmark spin-polarized DFT calculations for free Mn-salophen molecule. The molecular structure of Mn-salophen is illustrated in the top panel of Figure 1a. It is clear that Mn-salophen has planar structure. The Mn-N and Mn-O is about 1.95 and 1.87 Å, respectively, which are very close to the experimental values [29]. Due to the unpaired electrons of Mn atom, the ground state of Mn-salophen is spin-polarized and the molecular magnetic moment (MM) is predicted to be 3.0 μB. According to the spin-density distribution, as shown in the middle panel of Figure 1a, it is clear that the total molecular MM are mainly contributed by Mn atom since the spin-density mainly localizes around Mn ion. The atomic MMs of Mn, and N and O atoms connecting to Mn atom is about 3.24, −0.06, and 0.04 μB, respectively. Although the total molecular MM is mainly contributed by Mn atom, the neighbouring N atoms and several C atoms also give small negative contribution to the total MM. The bottom panel of Figure 1a shows the molecular energy levels and the spatial profiles of the highest occupied molecular orbital (HOMO) and lowest unoccupied molecular orbital (LUMO) of the spin-up and spin-down electrons. Clearly, the electronic structures display dramatically different feature for two spin channels. The spin-up HOMO delocalizes and locates at −1.43 eV, while the LUMO at 0.49 eV localizes around salophen group. As for the spin-down channel, the HOMO locates at −0.33 eV and delocalizes on the main body of the molecule, while the LUMO at 0.34 eV localizes around Mn atom. Then the energy gap between the HOMO and LUMO is predicted to be 1.92 and 0.67 eV for the spin-up and spin-down electrons, respectively. These predicted theoretical results agree well with previous investigation [30].

**Figure 2:**
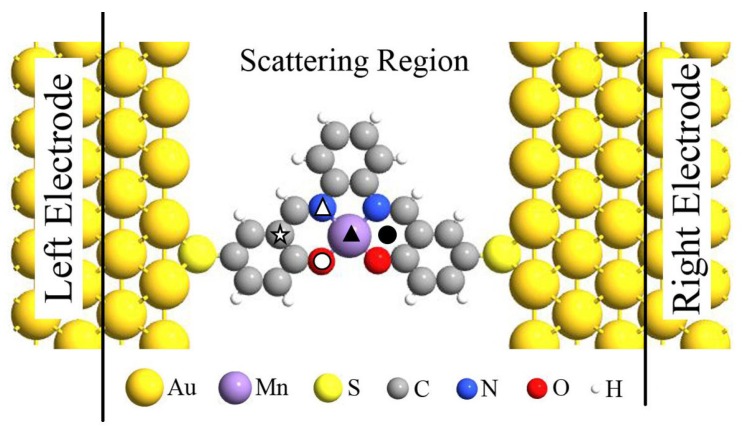
Schematic view of the proposed Mn-salophen molecular junction, here, the symbols stand for the examined adsorption sites.

Previous investigations have demonstrated that the spin-states of individual magnetic molecule (i.e., MnPc) can be reversibly controlled by single H atom adsorption [13] and spin-transport properties of Fe-porphyrin molecule can be significantly modified by adsorbing diatomic ligand gases (i.e., NO and CO) [18], now we turn to explore the chemical adsorption effect on the magnetic and spin-transport properties of Mn-salophen, which is sandwiched between two Au(100) electrodes with the stable hollow sites via the Au-S bonds. The proposed two-probe junction, as shown in Figure 2, can be divided into three parts: the left and right electrodes, and the central scattering region containing Mn-salophen, three and two surface layers of the right and left electrodes, respectively. Firstly, we determinate the energy favorable adsorption sites. The F, CO, and NO are initially placed on different possible positions in Mn-salophen, including the Mn-site, N-site, O-site, C-site, and the hollow site, labeled with different symbols in Figure 2. Then we optimize the adsorption height. According to the calculated total energies summarized in Table 1, we find that three different chemical adsorptions prefer to sitting on Mn atom in Mn-salophen molecular junction. Upon the chemical adsorption, the junction displays a slight geometric conformation. The Mn atom is slightly pulled out from the salophen plane, and the distance between Mn to F, to CO, and to NO is predicted to be 1.89, 1.77, and 1.63 Å, respectively. The relative short Mn-C, Mn-N, and Mn-F bond lengths suggest that F, CO, and NO molecules adsorb chemically on Mn-salophen.

Please note that the MM of Mn-salophen molecular junction (3.0 μB) is tuned to be 2.0, 1.0, and 0.0 μB via introducing F, CO, and NO adsorptions, respectively. The mechanism of MM tunability can be understood according to the atomic Mulliken population analysis and the calculated spin density (see the middle panel of Figure 1). Without the chemical adsorption, there are three unpaired *3d* electrons in Mn-salophen, and the corresponding molecular MM is 3.0 μB. Due to the large electronegative of F atom, one of three unpaired *3d* electrons of Mn ion transfers to F atom, the atomic MMs of Mn and F atoms are predicted to be 1.90 and 0.07 μB, respectively, then the molecular MM is 2.0 μB. As for CO adsorption, the lone pair electrons (namely two 5σ electrons) of CO are paired with two of unpaired *3d* electrons. The atomic MM of Mn, C and O atoms in CO is about 1.01, −0.08 and −0.02 μB, respectively, indicating that the Mn cation antiferromagnetically couples with CO molecule. Then the molecular MM is reduced to be 1.0 μB.

**Figure 3:**
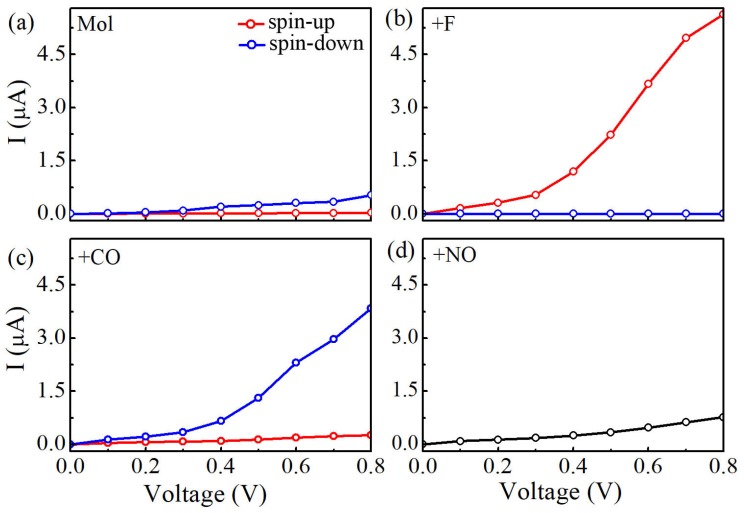
(**a**) Calculated currents of Mn-salophen molecular junction as a function of the applied bias voltage, (**b**–**d**) The I-V curves of Mn-salophen molecular junction with F, CO, and NO adsorptions. Here, the red and blue lines stand for the spin-up and spin-down electrons, respectively.

The obtained current-voltage (I-V) curves for four Mn-salophen molecular junctions under the bias voltage range from 0.0 to 0.8 V are plotted in Figure 3. In our calculations, the currents of the spin-up (I${}_{↑}$) and spin-down electrons (I${}_{↓}$) at each bias voltage are determined self-consistently under non-equilibrium condition by using the Landauer-Büttiker formula. Clearly, Here, the following important features can be easily observed in these calculated I-V curves:

(1) Without chemical adsorption, the currents through Mn-salophen molecular junction for the spin-up and spin-down electrons just show a little distinction. For example, at 0.4 V, the calculated I${}_{↑}$ is about 0.03 μA, while the I${}_{↓}$ is 0.09 μA, respectively. This observation agrees well with the previous report [30].

(2) Upon F adsorption on the central Mn atom, the current of the spin-down electrons (I${}_{↑}$) through Mn-salophen molecular junction is remarkably larger than that of the spin-up electrons (I${}_{↓}$). The conductance through molecular junction is dominated by the spin-up electrons. For example, at 0.8 V, the I${}_{↑}$ and I${}_{↓}$ is predicted to be 5.63 and 0.0028 μA, respectively. Here, we define the ratio of current as R(V)=$I_{↑}\left(V\right)/I_{↓}\left(V\right)|$ to quantify the current difference between the spin-up and spin-down channels under different bias voltages. The calculated value of R(V) varies from 800 to 2100 in the examined bias range. The predicted R(V) with large value implies that Mn-salophen junction with F adsorption can be used to design molecular spintronic device, such as spin filter.

(3) As for CO adsorption, we find that the conductance through molecular junction is dominated by the spin-down electrons. The calculated (I${}_{↓}$) through Mn-salophen molecular junction is remarkably larger than that of the spin-up electrons (I${}_{↑}$). The I${}_{↑}$ and I${}_{↓}$ is about 0.26 and 3.84 μA at 0.8 V, respectively, displaying again a nearly perfect spin-filtering. Remarkably, the conductive channel is switched from the spin-up electrons to the spin-down channel via adsorbing F and CO, respectively. Such a spin switch has been predicted in narrow zigzag graphene nanoribbon through placing square-shaped carbon tetragon [35].

(4) When NO connecting to Mn ion, the I${}_{↓}$ curve of Mn-salophen molecular junction completely coincides with that of the I${}_{↑}$. The reason is that the ground state of Mn-salophen molecular junction with NO adsorption is spin-restricted, and the corresponding molecular magnetic moment is quenched.

**Figure 4:**
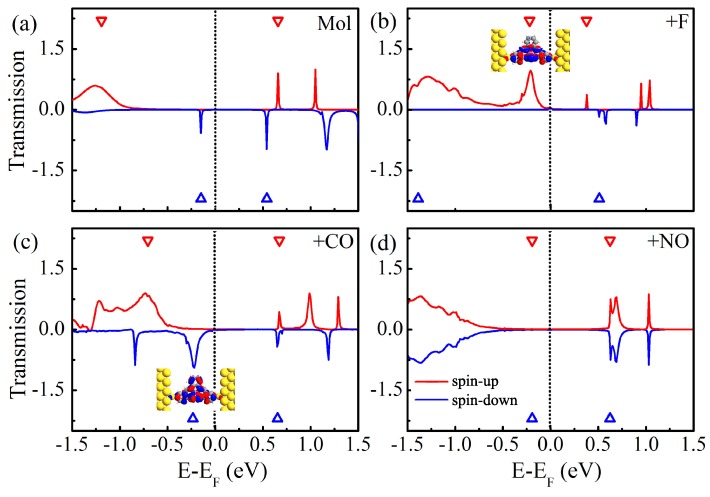
(**a**) The zero-bias spin-resolved transmission spectra of Mn-salophen molecular junction without chemical adsorption, (**b**–**d**) for Mn-salophen molecular junctions with F, CO, and NO adsorptions. Here, the short-dotted vertical lines stand for the Fermi level for clarity, while the red and blue lines stand for the spin-up and spin-down electrons, respectively, while the inserts in (**b**,**c**) stands for the spatial distribution of the perturbed HOMO of the spin-up and spin-down electrons.

To explore the nature of the above dramatically different characteristics of the I-V curves of four different molecular junctions, we calculate the zero-bias spin-polarized transmission spectra, and plot them in Figure 4. Here, the eigenvalues of the molecular projected self-consistent Hamiltonian are labeled with the empty triangles. For clarity, the average Fermi level is set as zero, and the conductances at the Fermi level are summarized in Table 2. As for Mn-salophen molecular junction without chemical adsorption, we obtain the very similar results presented in the previous report [30]. There are two narrow and sharp transmission peaks lying at −0.15 and 0.54 eV for the spin-down electrons, while there are two small transmission peaks locating at 0.66 and 1.05 eV for the spin-up electrons, respectively. Please note that a broad transmission peak at −1.26 eV does not give any contribution to the current within the range of integration since it locates far away from the Fermi level. These observations can be easily used to understand why the currents through Mn-salophen molecular junction for two different spin channels show only a little distinction.

Upon F adsorption, the overall features of transmission spectra of Mn-salophen molecular junction significantly changed, as shown in Figure 4b. It is clear that around the Fermi level the transport properties display remarkably different behavior for two spin channels. The transport property is dominated by the tail of the transmission peak of the spin-up electrons locating at −0.21 eV, which is contributed by the perturbed HOMO, which is plotted as the insert in Figure 4b. This low-bias transport properties governed by the spin-up electrons have been observed in molecular junctions based on FeN${}_{4}$ complexes [36,37].

Figure 4c shows the zero-bias spin-polarized transmission spectra of Mn-salophen molecular junction with CO adsorption. Clearly, contrast to the F adsorption case, the conductance through the junction is dominated by the spin-down channel via the tail of the transmission peak locating at −0.22 eV, which is contributed by the perturbed HOMO of the spin-down electrons (see the insert of Figure 4c). Actually, such a low-bias transport property governed by the spin-down electrons have been predicted for molecular junctions based on Fe-phthalocyanine [26], C${}_{28}$ [38], and Fe${}_{2}$ and Fe(II)-N${}_{4}$S${}_{2}$ complexes [21,23].

As shown in Figure 4d, the sharp peaks locating at 0.63 eV occur at the same positions for the spin-up and spin-down electrons in Mn-salophen molecular junction with NO adsorption, which causes the I-V curve of the spin-down electrons coincided with that of the spin-up electrons. Please note that this spin-up and spin-down transmission peaks, contributed by the perturbed HOMO, are rather far away from the Fermi level, exceeding the range of integration (i.e., [−0.4, 0.4 eV]) in Equation (2), and result in the small current through molecular junction for two spin channels, as shown in Figure 3c.

Next, we turn to analyze the spin-polarized electronic structures of Mn-salophen molecule with F, CO, and NO adsorption, as shown in Figure 1, since it is helpful to further understand the huge difference of electronic transport properties of these examined molecular junctions. It is clear that upon F adsorption, the energy gap between the HOMO and LUMO of the spin-up and spin-down electrons is predicted to be 0.59 and 1.89 eV, respectively, and the HOMO of the spin-up electrons locates close to the Fermi level. As for CO adsorption, the energy gap between the HOMO and LUMO of the spin-up and spin-down electrons is predicted to be 1.38 and 0.88 eV, and the HOMO of the spin-down electrons lies close to the Fermi level, as shown in Figure 1b,c. These results imply that the HOMO of the spin-up electrons provides the main transport channel for F adsorption case, and the main transport channel for Mn-salophen molecule with CO adsorption is contributed by the HOMO of the spin-down electrons. Clearly, these findings are confirmed by the calculated transmission spectra, as shown in Figure 4b,c.

**Figure 5:**
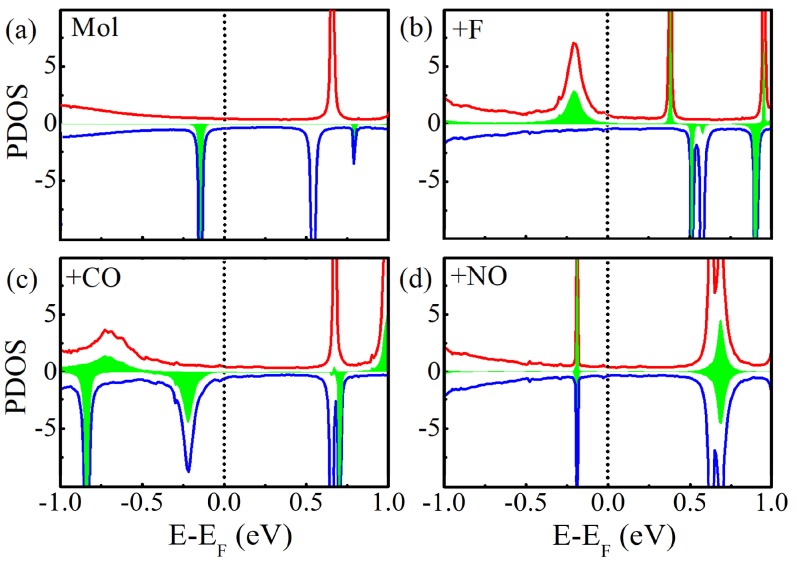
(**a**) The projected DOS of Mn-salophen molecule in junction without chemical adsorption, (**b**–**d**) for Mn-salophen with F, CO, and NO adsorptions. Here, the short-dotted vertical lines stand for the Fermi level for clarity, while the red and blue lines stand for the spin-up and spin-down electrons, respectively, and the PDOS of Mn cation is plotted with the filled regions with green color.

Figure 5 shows the projected density of states (PDOS) of Mn-salophen in the molecular junction. Here, the PDOS represents the discrete energy levels of free Mn-salophen molecule shifted and broadened due to the molecule-electrode coupling. It is clear that the transmission spectra shows the same qualitative shape as those of the projected PDOS curves, and the location of transmission peaks corresponds well the PDOS peaks. These significant transmission peaks originate from these broad PDOS peaks, in which the contribution from Mn action is observable. If the narrow PDOS peaks mainly originates from the PDOS of Mn cation, or no contribution from Mn cation in the narrow PDOS peaks, then they correspond to the narrow and small transmission peaks. For example, there is a broad PDOS peak of the spin-up electrons locating at −0.21 eV for Mn-salophen molecular junctions with F adsorption, while for the CO adsorption case, a broad PDOS peak of the spin-down electrons locates at −0.19 eV. Clearly, their positions agree well with the transmission peaks (see Figure 4), and can be used to understand why the low-bias transport properties are governed by the spin-up and spin-down electrons for F and NO adsorptions, respectively.

In general, standard GGA functionals (i.e., PBE) cannot predict correct HOMO-LUMO gaps for metal-containing molecules and some corrections, such as GGA+U, is necessary. [39,40] Here, we perform test DFT+U (U is set to be 2.5 eV for Mn atom). Fortunately, the DFT+U calculations qualitatively do not change the results given by the PBE functional. The position and shape of the transmission peaks just change slightly.

## 4. Conclusions

In summary, combining extensive density functional theory calculations with non-equilibrium Green’s function method, we explore the spin states and spin-transport properties of Mn-salophen sandwiched between Au electrodes. We find that the molecular MM of Mn-salophen can be effectively tuned via chemical adsorption. Without chemical adsorption, the currents of the spin-up electrons through Mn-salophen molecular junction is close to that of the spin-down electrons. Remarkably, theoretical results clearly reveal that the conductive channel can be switched from the spin-up electrons to the spin-down electrons via adsorbing F and CO, respectively, and the corresponding two Mn-salophen molecular junctions display nearly perfect spin-filtering effect. The observed spin-filtering effect and the switching between the spin-up and spin-down channels via chemical adsorption indicates that Mn-salophen holds potential applications in molecular spintronic devices.

## Figures and Tables

**Table 1:** Adsorption energies (in eV) on different positions.

Position	Mn-Site	N-Site	C-Site	O-Site	Hollow Site
F case	0.0	2.08	2.33	2.34	2.12
CO case	0.0	1.17	1.37	1.41	1.18
NO case	0.0	3.35	3.84	3.72	3.31

**Table 2:** Conductances (in G${}_{0}$) at the Fermi level for four examined molecular junctions.

Junction	Mn-Salophen	+F	+CO	+NO
spin-up	9.3 × 10${}^{-4}$	6.8 × 10${}^{-2}$	1.4 × 10${}^{-2}$	4.9 × 10${}^{-3}$
spin-down	2.1 × 10${}^{-3}$	5.7 × 10${}^{-5}$	5.4 × 10${}^{-2}$	4.9 × 10${}^{-3}$
